# Review of the Correlation of LAT1 With Diseases: Mechanism and Treatment

**DOI:** 10.3389/fchem.2020.564809

**Published:** 2020-10-20

**Authors:** Jingshun Zhang, Ying Xu, Dandan Li, Lulu Fu, Xueying Zhang, Yigang Bao, Lianwen Zheng

**Affiliations:** Reproductive Medical Center, Department of Obstetrics and Gynecology, The Second Hospital of Jilin University, Changchun, China

**Keywords:** cancer, immune diseases, insulin resistance, mTOR, MYC, LAT1

## Abstract

LAT1 is a member of the system L transporter family. The main role of the LAT1 is to transport specific amino acids through cell membranes to provide nutrients to cells and participate in several metabolic pathways. It also contributes to the transport of hormones and some drugs, which are essential for the development and treatment of some diseases. In recent years, many studies have shown that LAT1 is related to cancer, obesity, diabetes, and other diseases. However, the specific mechanism underlying the influence of LAT1 on such conditions remains unclear. Through the increasing number of studies on LAT1, we have obtained a preliminary understanding on the function of LAT1 in diseases. These studies also provide a theoretical basis for finding treatments for LAT1-related diseases, such as cancer. This review summarizes the function and mechanism of LAT1 in different diseases and the treatment of LAT1-related diseases. It also provides support for the development of novel and reliable disease treatments.

## Introduction

Although the main function of amino acids is participant in the production of protein, certain amino acids also play biological roles. For example, glutamine and glycine are necessary for nucleotide biosynthesis in essential cell proliferation processes. Serine has a key function as a one-carbon component that is indispensable to nucleotide synthesis and DNA methylation. Leucine and glutamine are vital signaling molecules and activators in several metabolic pathways (Bhutia et al., 2015). In mammals, the transmembrane transport of amino acids is regulated by multiple amino acid transporters (AAT), which are mainly located in the plasma membrane or intracellular compartments, such as Golgi apparatus, lysosomes, and mitochondria; these AAT promote the transmembrane exchange of amino acids or other substrates (Broer, 2002). Among AAT systems, the system L transporter family is one of the major plasma membrane transporter system. This family consists of four transporters: L-type AAT 1 (LAT1), coding by solute carrier family 7 member 5 (SLC7A5); L-type AAT 2 (LAT2), coding by solute carrier family 7 member 8 (SLC7A8); L-type AAT 3 (LAT3), coding by solute carrier family 43 member 1 (SLC43A1); L-type AAT 4 (LAT4), coding by solute carrier family 43 member 2 (SLC43A2) (del Amo et al., 2008). LAT1 is composed of 507 amino acids, contains 12 transmembrane regions, and has a molecular mass of 55,010 Da (Kanai et al., 1998; Mastroberardino et al., 1998). Its coding gene, SLC7A5, is located at 16q24.2 and contains 39,477 nucleotides and 10 exons. SLC7A5 belongs to the SLC7 family included in the larger APC (Amino acid–polyamine–organocation) superfamily. The SLC7 family consists of 15 members, two of which are pseudogenes. The 13 encoded proteins are classified in two subgroups: the cationic amino acid transporters and the light subunits (LATs) of the heterodimeric amino acid transporters (Scalise et al., 2018). LAT1 usually combines with 4F2 cell-surface antigen heavy chain (4F2hc; coding by solute carrier family 3 member 2, SLC3A2), a type II membrane glycoprotein that forms a heteromeric AAT complex via disulfide bonding; this complex is important for the stability of LAT1 and for localization to the plasma membrane (Yanagida et al., 2001; Verrey et al., 2004).

As a member of the system L transporter family, the main function of the LAT1 protein is to help specific amino acids pass through the cell membrane to provide nutrients to cells and participate in some metabolic pathways. The effect of LAT1 on human metabolism depends on its capability to identify specific amino acids and hormones as major physiological substrates and several drugs as non-physiological substrates (Scalise et al., 2018). LAT1 is a sodium-independent transporter and mainly responsible for the transport of branched-chain amino acids (BCAAs; e.g., valine, isoleucine, and leucine) and bulky amino acids (e.g., phenylalanine, tyrosine, tryptophane, asparagine, histidine and methionine). It is independent of any transmembrane ion gradient and is a compulsory exchanger, indicating that the flow of an amino acid into cell through LAT1 is forced to couple with the outflow of another amino acid (Bhutia et al., 2015). LAT1 also contribute to the transport of the hormones like T3, T4 and drugs, such as the dopamine precursor L-DOPA, melphalan, methyldopa, and gabapentin, which are essential for the development and treatment of some organs, particularly the brain, as well as the metabolic and pharmaceutical control of all tissues throughout life (Cornford et al., 1992; Friesema et al., 2001; Uchino et al., 2002; del Amo et al., 2008). Previous research showed that the expression of the LAT1 gene varies in accordance with tissue and cell type. LAT1 is highly expressed in mouse and human brains, testes, ovaries, pancreatic islets, and placentas but expressed at low levels in the lungs, hearts, and liver. It may provide nutrient substances for the normal function of these organs and cells. Although systemic capillary endothelial cells do not express LAT1, LAT1 is highly positively expressed in capillaries corresponding to the blood–brain barrier (BBB), blood follicles, and blood–retinal barrier (BRB) (Yanagida et al., 2001; Asano et al., 2007). Systemic homozygous LAT1 gene deletion can cause embryonic lethality among LAT1-knockdown embryos without apparent defects at day 8.5 of embryonic development. Soon after further embryonic development, the heads of the mutant embryos shrink and their trunks and tails shorten. These effects become serious at day 10.5 of embryonic development. Finally, no living LAT1-knockdown embryos are found at day 11.5 of embryonic development (Ohgaki et al., 2017). Mice lacking the domain required for interaction with LAT1 in SLC3A2 also fail to survive in the early stage of embryonic development (Tsumura et al., 2003), an outcome that can be attributed to the harmful influence of the lack of LAT1 transport function during mouse embryonic development after implantation (Sato et al., 2011). These phenomena explain the importance of the proper expression of LAT1 for embryonic development.

## LAT1-Related Diseases

Cell growth and proliferation rely heavily on the acquisition of exogenous amino acids, which are mainly used in proteins, nucleic acids, lipids, and adenosine triphosphate (ATP) biosynthesis (Wang and Holst, 2015). Therefore, AAT malfunction impairs the whole-body dynamic balance and causes a variety of human diseases, including cancer, insulin resistance, diabetes, immune diseases, inflammation, and so on. Although the specific mechanism of influence is not clear, it can also provide us with some new directions for the treatment of these diseases.

### LAT1 and Cancer

The proliferation of cancer cells is highly dependent on nutrients, in particular, some essential amino acids (EAAs) are essential. Their uptake of amino acids through the cell membrane is strictly controlled by membrane transporters. These transporters are usually increased in cancer cells relative to in normal cells to satisfy the substantial intake of amino acids during tumor progression (Wang and Holst, 2015). Numerous studies have suggested that LAT1 expression in cancerous tissues is higher than that in normal tissues and LAT1 expression is correlated with the growth and proliferation of cancer cells. These data are collected across a large number of cancers, showing in the Table 1. All previous studies indicated the importance of LAT1 in supporting the rapid growth and proliferation of cancers (Kim et al., 2004; Kaira et al., 2008a, 2009a, 2011a, 2012, 2013a; Ichinoe et al., 2011; Toyoda et al., 2014; Namikawa et al., 2015; Honjo et al., 2016; Hayase et al., 2017; El Ansari et al., 2018). For example, LAT1 expression in 53 ovarian cancer tissues was detected and compared with those in five normal ovarian tissues and five benign ovarian tumor tissues. LAT1 is obviously increased in various human epithelial ovarian cancers and ovarian cancer cell lines and is mainly located in the plasma membrane of ovarian cancer cells. LAT1 expressions in the ovarian cancer cell lines SKOV3, IGROV1, OVCAR3, and A2780 and the normal ovarian epithelial cell line ISOE397 are 22, 29, 24, 11, and 10 times higher than that in the normal ovary. LAT1 levels in ovariectomized tissues are also distinctly upregulated compared with those in benign ovarian tumors. The increased level of LAT1 may be associated with the proliferation and invasion capability of ovarian cancer cells (Kaji et al., 2010). Moreover, LAT1 expression is significantly higher in breast cancer than in normal breast tissue and more obvious in advanced breast cancer, indicating that LAT1 can act as a potential therapeutic target (El Ansari et al., 2018). By contrast, the inhibition or knockout of LAT1 expression by inhibitors and small interfering RNA (siRNA) can reduce the migration and invasion of cholangiocarcinoma cells *in vitro*, suggesting that the transport function of LAT1 is crucial for providing necessary nutrition to metastatic cancer cells (Janpipatkul et al., 2014).

**Table 1:** Expression of L-type amino acid transporter 1 (LAT1) in different tumor types.

**Cancer type**	**Expression**	**References**	**Meaning**
Tongue cancer	↑	Kaira et al., 2008b; Toyoda et al., 2014	An independent prognostic factor
Oral squamous cell carcinoma	↑	Kim et al., 2004	A more specific indicator of tumor progression
Esophageal squamous cell carcinoma	↑	Honjo et al., 2016; Hashimoto et al., 2017	The prognostic biomarker and therapeutic target
Thymic epithelial tumor	(+)	Kaira et al., 2009a	The immunohistochemical marker for carcinomas
Breast cancer	↑	Kaira et al., 2008b; El Ansari et al., 2018	An independent risk factor
Malignant pleural mesothelioma	↑	Kaira et al., 2011a	Associated with poor outcome
Lung tumor	↑	Kaira et al., 2008a; Rosilio et al., 2015	Associated with lymph node metastasis and poor outcome
Gastric carcinoma	↑	Ichinoe et al., 2011	An independent prognostic factor
Hepatocellular carcinoma	↑	Namikawa et al., 2015	An independent and significant prognostic factor
Pancreatic cancer	↑	Kaira et al., 2012	A significant prognostic factor
Biliary tract cancer	↑	Kaira et al., 2013a; Yanagisawa et al., 2014; Yothaisong et al., 2017	A significant independent predictor
Colorectal cancer	↑	Kaira et al., 2008b; Hayase et al., 2017	Associated with cancer cell proliferation via the mTOR pathway
Bladder tumors	↑	Xie et al., 2013	An independent prognostic factor
Prostate cancer	↑	Wang et al., 2011	Expression when hormone ablation and in metastatic lesions
Clear cell renal cell carcinoma	↑	Betsunoh et al., 2013	Related to the invasive and progressive potential
Malignant glioma cells	↑	Nawashiro et al., 2006; Kobayashi et al., 2008; Haining et al., 2012	The molecular target in glioma therapy
Endometrial carcinoma	↑	Marshall et al., 2016	An effective therapeutic strategy
Ovarian clear cell carcinoma	↑	Sato et al., 2019	Associated with poor prognosis and chemoresistance
Cutaneous melanoma	↑	Shimizu et al., 2015	An independent prognostic factor
Bone tumors	↑	Koshi et al., 2015	
Soft tissue tumors	↑	Kaira et al., 2008b; Koshi et al., 2015	
Lymphoblastic leukemia	↑	Rosilio et al., 2015	An efficient broad-spectrum adjuvant approach

*↑ means increased expression; (+) means positive expression*.

LAT1 level in tumors was recently proven to be an independent prognostic indicator (Nakanishi et al., 2006; Imai et al., 2009; Kaira et al., 2009b; Sakata et al., 2009; Ichinoe et al., 2011). In mice, the tumors produced by strongly LAT1-positive cells are larger than those produced by cells expressing low LAT1 levels (Salisbury and Arthur, 2018). The high expression of LAT1 in ovarian cancer has been verified to be associated with poor prognosis. The results of multivariate analysis showed that high LAT1 levels can serve as an independent variable that is suggestive of poor prognosis and FIGO stage III/IV (vs. I/II) in patients with ovarian carcinoma (Sato et al., 2019). The Kaplan–Meier analysis of patients with adenoid cystic carcinoma revealed that the total survival time of patients with low LAT1 levels is significantly longer than that of patients with high LAT1 levels, signifying that LAT1 is a predictive indicator of the poor prognosis of patients with adenoid cystic carcinoma (Kaira et al., 2013b). T-cell acute lymphoblastic leukemia (T-ALL) is a highly invasive disease that involves thymocyte transformation (Aifantis et al., 2008). The survival rate of mice with LAT1-silenced T-ALL tumors is better than that of mice with LAT1-expressing T-ALL tumors (Grzes et al., 2017). The relationship of the level of LAT1 with the malignant degree and prognosis of lung neuroendocrine tumors was investigated. The study included 21 cases of large cell neuroendocrine carcinomas (LCNEC), 13 cases of small-cell lung cancers, 5 cases of atypical carcinoids, and 10 cases of typical carcinoids. The results indicated that the level of LAT1 in LCNEC is positively correlated with lymph node metastasis and poor patient prognosis, and the expression of LAT1 increases as the malignancy of neuroendocrine tumors increases. Therefore, LAT1 is closely related to lymph node metastasis and poor prognosis in patients with lung neuroendocrine tumors (Kaira et al., 2008a). The level of LAT1 is also an important prognostic indicator in patients with pancreatic cancer. Among 97 patients with pancreatic ductal adenocarcinoma, ~53% have an upregulated level of LAT1, which is positively related to cancer cell proliferation, tumor angiogenesis, and disease progression (Kaira et al., 2012; Yanagisawa et al., 2012). Endometrial cancer is a major gynecological cancer in developed countries, and endometrial adenocarcinoma is its most common subtype, representing ~80% of all cases (Jemal et al., 2011). A Western diet is believed to be a factor that influences anomalous cell proliferation by providing an excess supply of animal proteins, especially BCAAs, such as leucine (O'Connell, 2013). LAT1 in serotypes and endometrioid endometrial adenocarcinomas is significantly upregulated relative to that in the normal endometrium. Patients with endometrial adenocarcinoma with the highest level of LAT1 have the lowest disease-free survival rate. LAT1 gene knockout significantly reduces leucine uptake by 51–58% and reduces cell growth in endometrial carcinoma cell lines. These results indicated that LAT1 is extremely essential to the regulation of leucine uptake and cell growth in endometrial cancer cells and the progression of endometrial carcinoma (Marshall et al., 2016). All the above results confirmed that LAT1 is significantly correlated with the invasiveness and prognosis of various cancers. Thus, LAT1 is a potential index for predicting the prognosis of patients with cancer.

LAT1 has also been shown to be associated with chemotherapy resistance. An analysis of the chemotherapy response of ovarian cancer revealed that several genes, including LAT1, can be critical factors leading to chemoresistance. LAT1 is a solute carrier protein that may regulate the absorption of drugs by cancer cells, thus regulating chemotherapy response. Therefore, LAT1 may be a novel target for improving the effect of chemotherapy on ovarian cancer (Cheng et al., 2010). LAT1 is also an indicator antibody in the endocrine therapy of breast cancer, where its high level may serve as a recurrence factor of estrogen-receptor positive breast cancer (Bartlett et al., 2010). LAT1 have a key role in the diverse periods of prostate cancer development. LAT1 is mainly expressed in the androgen-insensitive prostate cancer cell line PC-3. LAT1 levels is low in normal or benign prostate tumor tissues. With the transformation of hormone-sensitive prostate cancer to hormone-refractory prostate cancer, such as in castraton-resistant metastatic cancer tissue, the level of LAT1 is significantly upregulated. These results suggest that the increased level of LAT1 contributes to hormone-refractory prostate cancer and LAT1 may be a feasible target for the treatment of advanced prostate cancer (Wang et al., 2011, 2013). With the increasing number of cancer treatment methods, such as surgery, chemotherapy, radiotherapy, and other combined applications, the cure and survival rate of patients with cancer have increased significantly. However, chemoresistance has become a difficult problem encountered in current cancer treatment processes. Targeting LAT1 to improve the chemoresistance of patients with cancer may be another great progress in medical treatment.

### LAT1 and Insulin Resistance

Amino acids are increasingly considered as the regulators of nutrient processing (Lynch and Adams, 2014). For example, they regulate blood glucose levels through their interaction with insulin signals. The membrane transporters of amino acids were recently shown to be a key part of this regulatory process. Amino acids that are regulated by transporters can promote insulin secretion and regulate insulin signals in different tissues. Glucose also regulates the expression of LAT1. In patients with diabetes, an increase in blood glucose levels will reduce LAT1 expression and, subsequently, muscle reduction (muscular atrophy) (Yamamoto et al., 2017). By contrast, glucose deficiency upregulates LAT1 expression in the retina. The inner BRB is a delicate, compact junction of retinal capillary endothelial cells; it protects nerves and the retina from the effects of circulating blood and nourishes two-thirds of the cells in the retina (Hosoya and Tomi, 2005; Hosoya and Tachikawa, 2009). LAT1 is expressed in the internal BRB and provides EAAs to the neuroretina and retinal endothelial cells (Tomi et al., 2005). It is the major transporter of BCAAs in many non-epithelial cells and is directly involved in insulin secretion through leucine accumulation in the cytoplasm (Yoon, 2016). LAT1 is highly expressed in islets. The siRNA knockout of LAT1 in dispersed islet cells leads to a significant reduction in leucine-stimulated S6K1 phosphorylation, leucine-mediated insulin production, and islet cell proliferation. These effects indicate that LAT1 is necessary for the action of mammalian target of rapamycin complex 1 (mTORC1) and the mediation of islet β-cell signal and function; LAT1 may also be a new drug target for the therapy and prevention of type 2 diabetes (T2D) (Cheng et al., 2016). The realization that BCAAs, like leucine, is necessary for signal activation of the mTORC1 signal, a downstream molecule of insulin, has prompted many recent studies on the use of dietary leucine as an auxiliary therapy for obesity-associated insulin resistance. Doubling dietary leucine content can reverse numerous metabolite abnormalities and significantly improve glucose tolerance and the insulin pathway without influencing food intake or weight gain. Increasing dietary leucine greatly relieves hepatic steatosis and inflammation in adipose tissue. The above studies deduced that moderate changes in LAT1 levels can alter multiple metabolic pathways and insulin resistance (Macotela et al., 2011; Adeva et al., 2012). Insulin resistance is a common metabolic disorder, and its etiology is complex and not completely clear. If the role of LAT1 in the occurrence and development of insulin resistance is identified, then obesity, T2D, and other diseases associated with insulin resistance may be treated by modifying LAT1 expression or providing LAT1 substrates, such as leucine.

### LAT1 and Immune Diseases

The LATs, including LAT1, are potential immunosuppressive targets; For example, the high-affinity inhibitor of LATs, brasilicardin A is an effective immunosuppressant (Usui et al., 2006). T lymphocytes mediate adaptive immune reaction to antigens by producing and differentiating effectors. Therefore, activated T cells must be capable of upregulating their metabolism levels in time to meet the demands of normal reactions. In T cells, the regulation of LAT1 through the T-cell antigen receptor (TCR) is crucial for the effective functioning of immune response and the reprogramming of T-cell metabolism. For example, T-cell activation is necessary to upregulate the corresponding amino acid intake of cells through LAT1 to increase protein synthesis. Moreover, these amino acids are not only used to synthesize proteins (Wang and Green, 2012) but are also important for the CD8 cytotoxic T-cell (CTL) reaction. The response function of LAT1-null CD8^+^T cells to the cytotoxic effects of homologous antigens may be severely deficient (Sinclair et al., 2013). LAT1-specific knockout CD4^+^T cells can inhibit the inflammatory reaction induced by imiquimod. In the inflammatory model induced by imiquimod, the absence or inhibition of LAT1 will block the expansion of IL-17-secreting T cells and CD4^+^T cells as well as inhibit the production of the inflammatory cytokines IL-1β, IL-17, and IL-23. By contrast, IL-23 and IL-1β can stimulate the upregulation of LAT1 expression and promote the immune response. These results indicate that targeting LAT1 may be a potential immunosuppressive strategy to control skin inflammation induced by the IL-23/IL-1β/IL-17 axis (Cibrian et al., 2020). Histidine, a high-affinity substrate of LAT1, lays a foundation for explaining the following phenomena. The upregulated expression of LAT1 will increase histidine, obviously decrease the activation of nuclear factor-kappa B (NF-κB) and the stimulation of TNF-α, and control the level of E-selectin and the generation of IL-6 in human coronary arterial endothelial cells (HCAECs). The activation of NF-κB regulates the generation of various cytokines and the level of adhesion molecules involved in inflammatory diseases. The above study shows that in some cases, the increased expression of LAT1 also affects immunosuppression by affecting the histidine and NF-kB pathways in HCAECs (Hasegawa et al., 2012). LAT1 expression is reduced in an *in vitro* pancreatitis model and is associated with the role of LAT1 in acinar cells. Acinar cells accumulate amino acids through LAT1 and form a significant concentration gradient to synthesize digestive enzymes. However, LAT1 was not reduced in the *in vivo* pancreatitis model at RNA level and neither at protein level. They thought that LAT1 may be expressed in another cell population of which expression increases and masks the loss in the acinar cells in the *in vivo* model. Pancreatitis is a degenerative self-digestion process that is accompanied by the damage of exocrine acinar tissue and then by a regeneration process. Specific amino acid support has been confirmed to be beneficial to the cure of severe pancreatitis. Therefore, the reduction in amino acid levels caused by the decreased expression of LAT1 may be a reason for the aggravation of pancreatitis (Rooman et al., 2013). Because LAT1 provides cells with the most basic substance, amino acids. Whether in the process of anti-inflammation or in the occurrence and progress of inflammation, it may play an important role and maintain a dynamic balance. When the body is functioning normally, LAT1 may play an anti-inflammatory function. If the body is decompensated, LAT1 may accelerate the development of the disease and promote inflammation. At present, studies on the role of LAT1 in immune-related diseases remain scarce. However, LAT1 has been confirmed to be involved in immune and inflammatory responses. Thus, LAT1 may be used to explore a novel method for treating immune and inflammatory diseases.

### LAT1 and Neurological Disorders

LAT1 is a vital protein for organ growth and development because it is involved in the transport of eight of the nine EAAs to corresponding tissues (Hafliger and Charles, 2019). LAT1 is strongly expressed in the luminal and abluminal membranes of brain microvessel endothelial cells in all brain regions, including the cerebral cortex, cerebellum, hippocampus, gray matter, and white matter. It plays a key role in amino acid, hormone, and drug transfer across the BBB (Duelli et al., 2000). Reduced levels of LAT1 in the BBB are associated with the incidence and progression of Parkinson's disease (Ohtsuki et al., 2010). LAT1 is responsible for the transport of the dopamine precursor L-DOPA across the BBB. Therefore, reduced LAT1 expression leads to a decrease in L-DOPA distribution, which results in a corresponding reduction in dopamine production; this decrement is currently recognized as the cause of Parkinson's disease (Kageyama et al., 2000). Phenylketonuria (PKU) is caused by inherited phenylalanine hydroxylase (PAH) deficiency and is characterized by elevated blood levels of phenylalanine. The LAT1 is responsible for phenylalanine transport across the BBB. As phenylalanine excess in the brain leads to mental retardation in untreated patients with PKU, they deduced mutations of the LAT1 gene may be responsible for this situation (Bik-Multanowski and Pietrzyk, 2006). However, some scholars have analyzed the gene sequence variants in PKU patients. None of the identified variants changed the amino acid residues, nor did they lead to any formation of new splice sites, according to the consensus sequence for splice sites (Moller et al., 2005). Therefore, the exactly relationship between LAT1 gene mutations and PKU needs further study. The function of LAT1 in BBB can also be illustrated through the transport of tryptophan. Tryptophan is another EAAs and is a precursor of serotonin, melatonin, and vitamin B3, all of which are essential for the normal development of the nervous system (Walderhaug et al., 2002). Abnormal tryptophan levels affect behavioral and cognitive processes. A significant correlation exists between the anxiety index and tryptophan, as well as between T maze error, a recognized spatial working memory assessment tool, and LAT1. This relationship suggests that the decrease in LAT1 expression leads to a reduction in tryptophan transport through the BBB and affects neurological development (Asor et al., 2015). The change of LAT1 function in the BBB was also recently considered to be the molecular determinant of the autism spectrum disorder (ASD) (Al-Otaish et al., 2018; Smith et al., 2019). In a study, ninety-seven patients with ASD were screened by Sanger sequencing the genes, including LAT1. It was detected nine pathogenic variants in 11 of 97 patients (11.3%), and three among LAT1. Metabolic assays illustrated that such abnormalities affect the utilization of certain amino acids, particularly tryptophan and other LNAAs, with potential consequences on their transport across the BBB and their availability during brain development. Therefore, they concluded abnormalities in the LAT1 and are likely associated with an increased risk of developing ASD (Smith et al., 2019). The study by Tărlungeanu et al. reported two homozygous mutations (p. Ala246Val and p. Pro375Leu) in the LAT1 gene in patients with ASD and motor delay, and estimated the impact of these mutations on amino acid transport across BBB in a mouse model. The knockout of LAT1 in BBB endothelial cells in mice will result in atypical amino acid profiles, abnormal mRNA translation, and obvious neurological defects. The levels of leucine and isoleucine were reduced and the levels of histidine were increased significantly, and these abnormalities were rescued by intracerebroventricular injection of BCAAs. In addition, patients with deleterious homozygous mutations of the LAT1 gene in their BBBs will show some symptoms of autism characteristics and motor delay (Zheng et al., 2009; Tarlungeanu et al., 2016). These findings suggest potential dysfunction of LAT1 in individuals with ASD. Therefore, LAT1 can be used not only for amino acid delivery but also for the targeted treatment of brain and nervous system diseases.

It is a great challenge for the treatment of central nervous system (CNS) diseases, especially for neurodegenerative diseases, to deliver therapeutic drugs to brain lesions. In order to overcome this problem, various methods of drug delivery in the brain have been designed (Dong, 2018). The development of prodrugs similar with endogenous substrates of influx transporters, which are selectively expressed on the BBB and brain parenchyma cell membrane is a promising way of drug administration. After the prodrug mediated by transporter passes through the BBB and cell membrane barrier, the patent drug will be released at the target site of its brain parenchyma cells, so that the drug has a higher therapeutic concentration in the focus and improves the therapeutic effect (Puris et al., 2019a). Therefore, some scholars have developed LAT1-targeting prodrugs, which can significantly improve the cellular and brain uptake of several parent drugs, such as anti-inflammatory agent ketoprofen (Gynther et al., 2008), anti-epileptic drug valproic acid (Gynther et al., 2016a), anti-parkinsonian prodrug of dopamine(Thiele et al., 2018), investigational immunosuppressive perforin inhibitors (Gynther et al., 2016b), and natural phenolic antioxidant ferulic acid (Puris et al., 2019b). In a recent study, six LAT1-utilizing prodrugs were studied. All prodrugs were accumulated into the brain cells comparably and most cases even more effectively than their parent drugs. And in order to confirm that the cellular uptake these drugs were mediated via LAT1, the uptake of prodrugs was studied in the absence and the presence of LAT1-inhibitor (Huttunen et al., 2016). The result suggested that LAT1 -inhibitor has been able to reduce the uptake of the prodrugs in human breast cancer (MCF-7) or retinal pigmented epithelial (ARPE-19) cells by 65–93% (Puris et al., 2017, 2019b). Therefore, they concluded LAT1 can be utilized to increase the cellular uptake of drugs into the brain parenchymal cells by using a LAT1-prodrug approach (Huttunen et al., 2019). In another study suggested the LAT1-utilizing prodrug of ketoprofen achieved a higher extent of intracellular distribution from brain extracellular fluid (ECF) than the parent drug, ketoprofen. Meanwhile, the LAT1-utilizing prodrug of ketoprofen had no effect on the protein expression of LAT1 and CD98 subunit of the transporter in crude membranes of mouse and rat brain slices (Puris et al., 2019a). This study suggests that the LAT1-utilizing prodrug method may be a promising way to deliver drugs to the inner septum of brain parenchyma cells. In short, the above researches highlight the importance of the role of LAT1 in intra-brain targeted drug delivery which can lead to improved efficacy and safety of neuroprotective drugs within the brain.

### LAT1 and Other Diseases

In placentas, chorionic trophoblast cells differentiate into syncytiotrophoblast cells through cell fusion and maternal–fetal separate circulation. LAT1 is expressed on the maternal and fetal surfaces of the syncytiotrophoblast and plays a key part in the exchange of nutrient substances across the placental barrier (Ohgaki et al., 2017). LAT1 expression has been suggested to reduce in intrauterine growth restriction (IUGR), indicating that maternal nutrition to the fetus through the placental barrier is inadequate. Fetuses born with IUGR also have a significantly increased risk of suffering cardiovascular and metabolic diseases when they grow up (Pantham et al., 2016). Maternal obesity may be responsible for the increased expression of LAT1 in the placenta and for subsequent fetal overgrowth. Hence, the importance of normal LAT1 expression in placentas is further emphasized (Rosario et al., 2015). Given that the participation of LAT1 in many other diseases has not been found so far, the role of LAT1 in diseases should receive additional attention in the future.

## Mechanisms of LAT1 in Diseases

As we summarized before, LAT1 is involved in the occurrence and development of many diseases, but how does it work? At present, there have been several studies on the mechanism of LAT1, although it is not clear, but it also provides us with some ideas to understand the occurrence of these diseases from another point of view.

### LAT1 and Mammalian Target of Rapamycin

LAT1 is located on cell surfaces and interacts with the intracellular nutrient signaling pathways (e.g., mTORC1 pathway) that regulate cell metabolism (Hundal and Taylor, 2009). Mammalian target of rapamycin (mTOR) is a member of the phosphoinositide 3 kinase-related kinase family, which has the catalytic activity of protein serine threonine kinase. mTOR is mainly present in two kinds of complexes in cells: mTORC1 and mTORC2. mTORC1 consists of mTOR complexed with mLST8, RAPTOR, PRAS40, and DEPTOR; it activates S6 kinase and inhibits the eIF-4E binding protein (Hara et al., 1997). This complex harmonizes signals from pressure, energy state, and oxygen supply to coordinate cell autophagy, growth, and protein synthesis. mTORC2 is composed of mTOR complexed with mLST8, RICTOR, and mSin1; this complex is mainly regulated by growth factors and affects cell growth and metabolism (Sarbassov et al., 2004; Jacinto et al., 2006; Sancak et al., 2010; Arnsburg and Kirstein-Miles, 2014). In an environment rich in amino acids, mTOR can stimulate and regulate protein synthesis while inhibiting autophagy. If extracellular amino acids are restricted, then autophagy will be activated to recycle other intracellular components as substitute sources of amino acids (Nicklin et al., 2009; Saxton and Sabatini, 2017). LAT1 sometimes enters lysosomal membranes through lysosomal-associated transmembrane protein 4b and regulates leucine exchange into the lysosome (Milkereit et al., 2015). After entering the lysosome, leucine can regulate the activation of lysosomal membrane H+ATPase (V-ATPase), which is an important component of the mTORC1 activator complex on the lysosomal surface, thereby promoting the activation of mTORC1 (Zoncu et al., 2011). Meanwhile, LAT1 expression is adjusted by transcription factor 4 (ATF4) by signaling in an mTORC1-dependent manner. Cell nutrition insufficiency caused by amino acid deficiency activates ATF4, which guides amino-acid transport into cells. Elevated ATF4 levels upregulate LAT1, resulting in an increased uptake of isoleucine or leucine and therefore activating mTORC1 and inhibiting autophagy. Recent studies have shown that the knockout of LAT1 or ATF4 can block amino acid uptake, prevent mTORC1 activation, and enhance autophagy (Chen et al., 2014). mTORC1 mainly regulates cell metabolism and protein synthesis. The abnormal activation of mTORC1 is related to various diseases. First, the mTORC1 signal accelerates tumor development by influencing various metabolic pathways to promote the growth, proliferation, and anti-apoptosis of cancer cells. Recent studies have suggested that human prostate cancer cell lines can upregulate the absorption of amino acids through LAT1, thereby promoting mTORC1 signal transduction and cell growth (Wang et al., 2011). The pharmacologic inhibition or knockout of LAT1 can inhibit the growth and mTOR signaling pathways of various tumor cell lines, such as human colon adenocarcinoma cells (LS174T and HT29), lung cancer cells (A549 and H1975), and renal carcinoma cells (786-O and A498) (Oda et al., 2010; Cormerais et al., 2016; Salisbury and Arthur, 2018). These outcomes emphasize that the overexpression of LAT1 is a frequently observed phenomenon in the process of mTORC1 pathway-associated cancer transformation. The activation of the hypoxia-inducible factor 2α (HIF-2α) pathway can likewise promote the expression of LAT1, thus increasing mTORC1 phosphorylation and activating mTORC1 in the process of cancer progression. Under the condition of oxygen deficiency, mTORC1 activity is generally restrained, which is a mechanism for saving energy through inhibiting mTORC1-related metabolism. The regulatory effect of HIF-2α on LAT1 expression provides a molecular mechanism for the increased level of LAT1 in cancer cells. Sequence analysis shows that the proximal promoter of human LAT1 contains two HIF-2α binding sites at −112 and −458. In renal carcinoma cells and normal liver and lung cells, HIF-2α binds directly to LAT1 through its DNA-binding region, thus participating in LAT1 gene expression and activating mTORC1 signal transduction (Elorza et al., 2012). Hypoxia-inducible factor 1α (HIF-1α) can stimulate angiogenesis and glycolytic enzyme transcription and is a downstream component of the mTOR pathway. HIF-1α may regulate the expression of LAT1, which has a notable correlation with HIF-1α in non-small cell lung cancer (Kaira et al., 2011b). Consequently, LAT1 is involved in cancer development and invasion through the mTOR pathway by providing nutrients and participating in oxygen regulation and autophagy. LAT1 can increase the intracellular supply of leucine and then activate the mTORC1 pathway, which is essential for regulating CTL differentiation, memory, and migratory capacity (Araki et al., 2009; Zheng et al., 2009; Powell and Delgoffe, 2010; Rao et al., 2010). In patients with rheumatoid arthritis (RA), LAT1 is upregulated in the synovium. The RNA interference method was used to study the effect of LAT1 gene inhibition on the synovial fibroblast-like cells (FLS) of patients with RA to explore its effect on RA. SiRNA interference with LAT1 decreases the phosphorylation of mTOR with its downstream target 4EBP1, uptake of leucine, and migratory capability of RA FLS. Cells treated with the inflammatory cytokine IL-17 stimulate LAT1 expression and participate in the LAT1-mediated migration of fibroblasts. By contrast, the promoting effect of IL-17 on LAT1 is neutralized by the application of the mTOR signal inhibitor temsirolimus or eIF4E inhibitors. SiLAT1 also significantly reduces IL-17-mediated leucine absorption and cell migration. In conclusion, LAT1 overexpression induced by IL-17 through the mTOR/4EBP1 pathway aggravates FLS migration in RA (Yu et al., 2018). Similar to amino acids that regulate the mTOR pathway, some hormones, such as insulin and insulin-like growth factor-1 (IGF-1), serve as essential upstream mediators of mTORC1 signaling. When insulin or IGF-1 receptor binds to a ligand, mTORC1 can be activated, which increases protein synthesis (Kandasamy et al., 2018). The dysregulation of LAT1 and mTOR is also related to obesity and T2D (Cohen and Hall, 2009). Mucosa-associated invariant T (MAIT) cells significantly upregulate their glycolysis rate in an mTORC1-dependent manner during activation. This behavior is crucial for the normal function of MAIT cells. MAIT cells isolated from obese adults all present glycolysis, mTORC1 signal, and LAT1 transport defects (O'Brien et al., 2019). Muscle-specific LAT1 knockout mice show slight insulin resistance and reduced mTORC1 pathway activation in their skeletal muscle after being fed a high-protein diet (Taylor, 2014). Moreover, compared with control mice, muscle-specific LAT1 knockout mice exhibit slightly increased insulin resistance in a variety of dietary patterns. This characteristic suggests that insulin signaling is related to the expression of LAT1 in muscle tissue and may be associated with the potential activation abilities of leucine, the concentration of BCAA, and the flux of LAT1 substrates for the mTORC1 pathway (Poncet et al., 2014). As can be concluded from the above results, LAT1 is involved in cancer, immune response, and insulin signal regulation through the mTOR pathway. However, the detailed mechanism of this involvement needs further study.

### LAT1 and MYC

MYC genes are among the oncogenes that were the first to be discovered, including c-Myc, n-Myc, and l-Myc. LAT1 not only participates in the activation of mTORC1 but is also associated with the expression of MYC in cancer cells. c-Myc is a vital positive mediator of LAT1. The LAT1 promoter has a typical c-Myc binding site (Blackwell et al., 1990), and the overexpression of this oncogene leads to an increase in LAT1 expression. siRNA decreases the expression of c-Myc, which leads to a decrement in LAT1 protein and mRNA levels and then causes obvious leucine absorption deficiency in pancreatic cancer cells. c-Myc is an essential transcription factor for LAT1 regulation (Hayashi et al., 2012). MYC upregulates the levels of tryptophan and tryptophan metabolites in the kynurenine pathway by inducing the expression of the tryptophan transporter LAT1 and increasing the expression of the enzyme arylformamidase (AFMID), which promotes the conversion of tryptophan into tyrosine. LAT1 and AFMID levels increase in colon cancer cells and tissues, and kynurenine levels in tumor samples are higher than those in the adjacent normal tissues of patients with colon cancer. Abnormal LAT1 expression regulated by MYC plays an important role in colon cancer (Venkateswaran et al., 2019). LAT1 and MYC can promote each other's expression and activity in Burkitt's lymphoma and neuroblastoma cells. In a study on the influence of LAT1 on the metabolic pathway of MYC, the results indicated that MYC promotes the expression of LAT1 and, after MYC silencing, LAT1 mRNA and protein levels decrease significantly. The inhibition of LAT1 expression can significantly reduce the growth of Burkitt lymphoma and neuroblastoma cells, suggesting that MYC-induced LAT1 is closely related to tumor cell proliferation (Yue et al., 2017). Subsequently, LAT1- and MYC-knockout Burkitt lymphoma cells were subjected to gene expression profiling. The changes in gene expression after LAT1 knockout are likely caused by the decrease in MYC protein expression in LAT1 silencing cells. These results indicate that LAT1 and MYC regulate Burkitt lymphoma cell proliferation together (Salisbury and Arthur, 2018). Thus, LAT1 may be involved in the onset and progression of tumors through its interaction with the oncogene MYC.

## Application of LAT1 in the Diagnosis

Auxiliary examination technology targeting LAT1 to detect tumors has gradually developed in recent years. The knowledge of LAT1 overexpression and substrate specificity has been used in the development of radiolabeled probes for cancer diagnosis. Labeling [^18^F] or [^11^C] in the basal structure of LAT1 allows positron emission computed tomography (PET) imaging of compounds accumulated in the tumor after administration. Thus, several amino acids based probes have been developed including (S)-2-amino-3-[3-(2-[^18^F]-fluoroethoxy)-4-iodophenyl]-2-methyl propanoic acid ([^18^F]-FIMP), L-3-[^18^F]-fluoro-α-methyl tyrosine ([^18^F]-FAMT), 6-[^18^F]-fluoro-L-3,4-dihydroxy-phenylalanine ([^18^F]-DOPA), L-[^11^C]-methyl-methionine ([^11^C]-MET) and O-(2-[^18^F]-fluoroethyl)-L-tyrosine ([^18^F]-FET) (Puris et al., 2020). For example, in the LAT1-specific PET probe, FAMT is specifically accumulated in the site of malignant tumors. FAMT does not appear in non-cancerous areas, such as sarcoidosis and inflammatory lesions. The accumulation level of FAMT in PET images is closely related to the expression level of LAT1 in malignant tumors (Nobusawa et al., 2013). Therefore, LAT1-specific probes can be used to distinguish tumors from granulomatous and inflammatory benign lesions. Moreover, FAMT-PET probes prove that the levels of LAT1 in cell membranes are specific to human cancers. These molecular probes have a strong affinity for LAT1 because of their α-methyl moieties. Given that α-methylated aromatic amino acids, such as α-methylphenylalanine, α-methyltyrosine, and α-methyldopa, specifically target LAT1 but have little influence on LAT2-regulated absorption, they are highly selective for LAT1 (Morimoto et al., 2008). Thus, given its excellent selectivity for LAT1, FAMT is used as an LAT1-specific probe that can be applied to find cancer through PET imaging (Kandasamy et al., 2018). The other probe [^11^C]-MET has high specificity in the detection, qualitative diagnosis of tumor because of its convenient synthesis, rapid and strong specificity, and has become the most commonly used radiolabeled method (Tsukada et al., 2006; Glaudemans et al., 2013). In the research of Okubo et al. indicated that uptake of [^11^C]-MET in human newly diagnosed gliomas was associated with the extent of LAT1 expression (Okubo et al., 2010). LAT1 targeted anticancer drugs have been used in boron neutron capture therapy (BNCT), mainly for patients with high-grade gliomas (Kawabata et al., 2009; Kankaanranta et al., 2011). BNCT is a kind of radiotherapy based on nuclear fission reaction. When ^10^B is irradiated by low-energy thermal neutron beam, high-energy alpha particle (^4^He^2+^) and recoil lithium (^7^Li) nucleus, the nuclear fission reaction occurs (Barth et al., 2005). The efficacy of BNCT depends on the accumulation of ^10^B in cancer tissue, which can be improved by LAT1-mediated transmission. Overall, these findings provide a new idea for specific non-invasive tumor location and targeted tumor injection treatment, which may lead toward a new trend of tumor diagnosis and treatment in the future.

## Application of LAT1 in the Treatment

In tumor therapy, many cytotoxic anticancer drugs exert an antitumor effect by interfering with the biosynthesis and function of nucleic acids. However, these anticancer drugs have cytotoxic effects on cancer and normal cells, thus causing adverse reactions, such as myelosuppression, gastrointestinal disorders, and renal dysfunction (Rosenberg et al., 1969; Capranico et al., 1990; Rowinsky et al., 1990). As mentioned before, LAT1 has broad application prospects as a biomarker for tumor diagnosis, a molecular target for chemotherapy, and a selective target for radiotherapy (Nawashiro et al., 2006; Kim et al., 2008; Kaira et al., 2009c). Given that knocking out LAT1 can inhibit tumor proliferation and invasion, some scholars have begun to explore the effect of LAT1 inhibitors on tumors. The production of LAT inhibitors mainly concentrates on compounds mimicking LAT substrates so they can compete for amino acid binding. For example, the leucine analog BCH (2-aminobicyclo- (Broer, 2002; Bhutia et al., 2015)-heptane-2-carboxylic acid) can inhibit amino acid uptake in tumor cells (Mastroberardino et al., 1998; Kim et al., 2002). With members of the LAT1 family sharing most substrates, BCH can be used to block LAT family members that are undesirable for application in clinical treatment (Wang and Holst, 2015). Amino acid transport assay, DNA fragmentation analysis, and terminal deoxynucleotidyl transferase-mediated dUTP nick end labeling (TUNEL) assay have been used to study the inhibitory effects and mechanism of BCH on cancer cells. BCH decreases the growth and L-leucine transport of KB human oral epidermoid carcinoma cells, Saos2 human osteosarcoma cells, and C6 rat glioma cells in a time- and concentration-dependent manner. Meanwhile, after BCH treatment, DNA ladder structures appear and TUNEL-positive cells increase, suggesting cancer cell death. Caspase is a family of proteolytic enzymes that is a key player of apoptosis. Caspase enzymes are synthesized into inactive protoenzymes and require the activation of proteolytic enzymes (Datta et al., 1997). Procapase-7 levels in cancer cells without BCH treatment are low and decreased in KB, Saos2, and C6 cells after BCH treatment. BCH treatment also promotes the proteolysis of procaspase-3 in KB and C6 cells (Datta et al., 1997). These results indicate that BCH inhibits LAT1 activity in cells and causes EAA loss, which leads to cancer cell apoptosis (Kim et al., 2008). BCH promotes cancer cell apoptosis by activating caspase and inhibits DNA synthesis by promoting the expression of the cyclin-dependent kinase inhibitors p21 and p27, thus playing a role in cytostatic and cytocidal influences on human malignant glioma cells with positive LAT1 expression (Kobayashi et al., 2008). The expression of LAT1 in human ovarian cancer cell lines SKOV3, IGROV1, A2780, and OVCAR3 is considerably higher than that in the normal ovarian epithelial cell line IOSE397. BCH significantly inhibits the proliferation, migration, and L-leucine uptake of OVCAR-3 cells (Kaji et al., 2010). BCH also decreases the phosphorylation of p70S6K, a downstream effector of mTOR, in SKOV3 and IGROV1 cells and significantly reduces the proliferation of these two cell lines. Finally, BCH increases the sensitivity of IGROV1 and A2780 cells to bestatin, an antiproliferative aminopeptidase inhibitor, and induces the inhibition of cancer cell proliferation. These effects suggest that combined therapy has a synergistic effect. Hence, LAT1 may be the target of combined antiproliferative aminopeptidase inhibitors in the treatment of ovarian cancer (Fan et al., 2010). Yamauchi et al. (2009) also suggested that BCH increases the antitumor capability of cisplatin in a head and neck squamous cell carcinoma cell line. However, if BCH acts on normal living cells, then the amino acids that are indispensable to protein synthesis and cell growth and metabolism may become deficient, leading to cell damage.

The treatment of cells with the LAT1 inhibitor BCH also decreases glucose levels and relieves insulin resistance. The mitochondrial membrane potential and ATP production of islet β cells exposed to high concentrations of glucose and free fatty acids are significantly reduced (El-Assaad et al., 2010). These effects are believed to promote the development of T2D by impairing β cell function and causing β cell apoptosis and are generally known as β cell glycolipidoxicity (El-Assaad et al., 2003; Poitout and Robertson, 2008). In an *in vivo* study, 7-week-old diabetic db/db mice received BCH and placebo treatment for 6 weeks. After treatment, all the mice were subjected to the intraperitoneal glucose tolerance test (IPGTT) and immunohistological examinations. The IPGTT revealed that the mice in the db/db control group exhibited significant glucose intolerance. Interestingly, the BCH treatment group presented significantly increased glucose tolerance and insulin secretion. Moreover, in the db/db control group, islets were destroyed, stained β cells decreased, and glucagon-positive α-cells that penetrated into the whole islets appeared. The morphology of the islet cells of the db/db mice treated with BCH was relatively normal, the number of α cells in islet cores was significantly reduced, the ratio of insulin-positive β cells among the total area of islets increased, and the percentage of β cells expressing caspase 3 decreased. These studies showed that BCH can enhance β cell function and avoid β cell apoptosis in diabetic db/db mice (Han et al., 2012). Another study showed that BCH increases insulin secretion induced by low glucose and high glucose (HG) by 1.8 and 1.2 times, respectively. Culturing β cells under HG conditions for a period of time can reduce insulin concentration, whereas BCH treatment can restore the HG-mediated decrease in insulin concentration. BCH also blocks the inhibition of glucose-stimulated insulin secretion induced by HG and the decrease of insulin gene expression induced by HG/palmitic acid (PA). BCH also has a concentration-dependent protective effect on HG/PA-induced DNA fragmentation. The C-Jun N-terminal kinase (JNK) pathway is an important regulator of endoplasmic reticulum (ER) stress induced by fatty acids. Glucose can enhance JNK phosphorylation during ER stress induced by PA and induce β cell apoptosis (Martinez et al., 2008; Bachar et al., 2009). BCH can reduce HG/PA-stimulated phospho-JNK levels with a concentration-dependent pattern. Furthermore, the long-term BCH treatment of mice can improve most of the metabolic changes caused by a high fat/high fructose (HF/HFr) diet. Numerous stress and inflammation-related signals, such as JNK, p38, pancreatic endoplasmic reticulum kinase (PERK), and NF-κB, have been proven to be related to insulin resistance and liver injury induced by a Western diet. A study suggested that BCH can prevent the HF/HFr-stimulated accumulation of adipose and the activation of stress and inflammatory pathways in liver tissue. For example, BCH reduces the level of phosphorylated JNK, phosphorylated PERK, phosphorylated p38, and phosphorylated NF-κB. BCH likewise decreases the expressions of TNF-α and IL-1β in the liver and the contents of alanine transaminase and aspartate transaminase in the liver collagen and plasma of HF/HFr mice. Compared with the control diet, the HF/HFr diet upregulates fasting blood glucose by ~43%. BCH treatment restores the elevated fasting blood glucose levels caused by HF/HFr to close to the blood glucose level of the control group and improves glucose tolerance. These results indicate that stress, inflammation, and toxicity pathways stimulated by long-term HF/HFr diet can be prevented by BCH, which can be used as a strategy to prevent non-alcoholic fatty liver disease and T2D caused by a high-calorie Western diet (Ruderman et al., 2010; Houtkooper et al., 2012; Han et al., 2016). The LAT1 inhibitor BCH can not only inhibit cancer cell proliferation and induce cancer cell apoptosis but also improve insulin resistance and relieve inflammation. However, BCH cannot be used in the clinic because of its low specificity and possible side effects. Thus, the discovery of an LAT1 inhibitor with similar effects as BCH has become an urgent problem that must be solved.

Several new compounds that can specifically inhibit LAT1 have been generated through synthetic chemistry and *in vitro* screening. These compounds include the novel tyrosine analog JPH203, (S)-2-amino-3(4-[(Bhutia et al., 2015) meth-oxy]-3,5-dichlorophenyl) propanoic acid, which is also called KYT-0353 and SKN103. SKN103 was synthesized based on the structure of T3 which displayed inhibite the proliferation of pancreatic cancer cells and squamous cell carcinoma cells (Kongpracha et al., 2017). JPH203 suppresses LAT1 through substrate competition (Yun et al., 2014). The microenvironment of the nude mouse system used for JPH203 therapeutic assessment is akin to that of human tumors, illustrating that JPH203 may have clinical efficacy in humans (Oda et al., 2010). Therefore, JPH203, a new LAT1-specific inhibitor, has become a potential tumor chemotherapy drug. Surprisingly, JPH203 does not damage normal murine thymocytes, lymphocytes, erythrocytes, platelets, bone marrow mature cells, stem cells, and early progenitors (Yun et al., 2014; Hayashi and Anzai, 2017). It may inhibit the activation of mTORC1 and the level of c-Myc in T-ALL and T-cell lymphoblastic lymphoma. JPH203 can also significantly inhibit the activity of T-ALL cells. It reduces the survival rate of T-ALL cells from mice and patients *in vitro* and inhibits the growth of T-ALL tumors in mice. Meanwhile, it has no effect on the viability of normal T cells or other types of blood cells (Rosilio et al., 2015). Other research showed that JPH203 inhibits leucine absorption and cell proliferation in human colon cancer-derived HT-29 cells and mouse renal proximal tubule cells. Moreover, the intravenous injections of 12.5 and 25.0 mg/kg JPH203 have a significant inhibitory effect on HT-29 tumors transplanted into nude mice, with the maximum inhibitory rates of 65.9 and 77.2%, respectively. However, weight gain, which is a symbol of security, has negligibly decreased. Thus, although JPH203 can obviously inhibit the growth of HT-29 cells *in vitro* and *in vivo*, it only results in slight weight loss (Oda et al., 2010). Given that the specific LAT1 inhibitor JPH203 has been proven to inhibit the growth of tumor cells and has little effect on other cells, it is expected to become a new antitumor drug in the clinic. However, the clinical use of JPH203 needs further clinical verification. Meanwhile, the effects of other LAT1 inhibitors on immunity, inflammation, and insulin-resistance-related diseases have not been studied yet and are thus new directions for future research.

In the past few years, the potential of LAT1 to enhance the entry of nanoparticles into cancer cells has been studied to treat cancer. In the studies of Li et al., nanoparticles were conjugated with glutamate as ligand for LAT1. The result showed that the antitumor efficiency of glutamic acid conjugated paclitaxel nanoparticles (SPG25 NPs) *in vitro* and *in vivo* was higher than that of unconjugated nanoparticles (Li et al., 2017). Similarly, in the other study of the same team, glutamate-conjugated docetaxel-loaded liposomes showed an increased cytotoxicity *in vitro* and higher accumulation in the brain compared to unconjugated liposomes (Li et al., 2016). In another words, Ong et al. found that the combination of levodopa and anisotropic gold nanoparticles (AuNPs) mediated selective photothermal ablation of breast cancer cells and chemosensitized cells (Ong et al., 2017). In conclusion, these recent studies suggest that LAT1 targeting nanoparticles may be a promising method to improve the efficacy of nanoparticles.

## Conclusion

**Figure 1:**
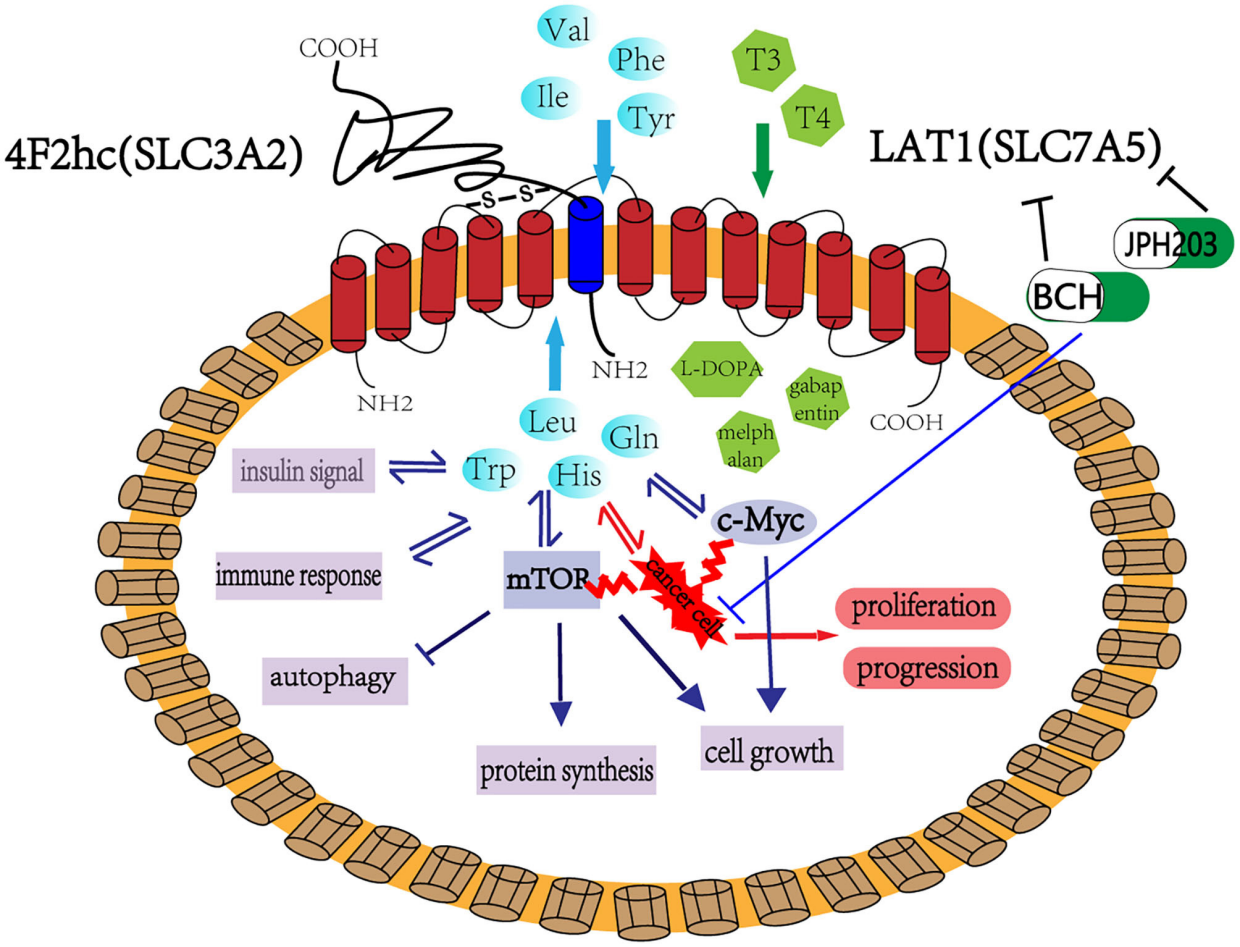
LAT1 binds with 4F2hc (SLC3A2), which are located on the plasma membrane. This complex is responsible for transporting amino acids, hormones, and some drugs. LAT1 regulates cell growth and metabolism through the mTOR and MYC pathway and participates in insulin signaling, immune response, and cell autophagy. LAT1 also plays an important role in tumor occurrence and development. BCH and JPH203 are the two main inhibitors of LAT1, which can inhibit the growth of tumor cells and has considerable application prospect in tumor treatment.

LAT1, an AAT, transports amino acids and regulates a variety of cell growth and metabolic processes. The main mechanisms related to LAT1 are illustrated in Figure 1. Fundamental research on LAT1 has made rapid progress since the importance of LAT1 in tumor cells was confirmed. LAT1 currently has broad application prospects as a biomarker for tumor and other diseases diagnosis, a molecular target for chemotherapy, and a selective target for diseases therapy. In recent years, many scholars worldwide have found highly effective and selective LAT1 inhibitors that have presented good anticancer effects in preclinical and clinical research. In particular, the latest phase of clinical trials has shown that JPH203 is effective for biliary tract cancer (Okano et al., 2020). The role of LAT1 has been gradually confirmed not only in tumors but also in inflammatory reactions, immune responses, insulin resistance, and other diseases. However, the mechanisms of the function of LAT1 in these diseases has not been clarified and the role of LAT1 inhibitors in diseases has not been studied. In the future, our task is to further confirm the role of several specific inhibitors of LAT1 in the treatment of various tumors and study its therapeutic effects on inflammation, immune-abnormal diseases, and insulin resistance to provide a new concept for the treatment of LAT1-related diseases.

## Author Contributions

JZ, YX, DL, LF, XZ, and YB performed literature searches and selected the studies and reviews discussed in the manuscript. The first draft of the manuscript was prepared by JZ. LZ performed subsequent amendments. JZ and YX revised the manuscript. All authors read and approved the final manuscript. All of the authors contributed to the conception of the review.

## Conflict of Interest

The authors declare that the research was conducted in the absence of any commercial or financial relationships that could be construed as a potential conflict of interest.
